# Leukemia Cutis in Acute Myeloid Leukemia: A Case Report

**DOI:** 10.7759/cureus.107112

**Published:** 2026-04-15

**Authors:** Amin Saied Sanosi Saied, Motaz Almahmood, Abdulrahman Al-Mashdali, Muhammad Ali Tariq, Mohamed Yassin

**Affiliations:** 1 Internal Medicine, Hamad General Hospital, Doha, QAT; 2 Internal Medicine, Tower Health Medical Group, Phoenixville, USA; 3 Internal Medicine, Hamad Medical Corporation, Doha, QAT; 4 Hematology, National Centre for Cancer Care and Research, Hamad Medical Corporation, Doha, QAT

**Keywords:** acute myeloid leukemia (aml), cutaneous myeloid sarcoma, gingival hyperplasia, leukemia cutis, myeloid sarcoma, skin biopsy

## Abstract

Leukemia cutis is an uncommon extramedullary manifestation of acute myeloid leukemia (AML) caused by infiltration of the skin by leukemic blasts. Its clinical morphology is highly variable and may mimic infectious, inflammatory, or other neoplastic dermatoses, making tissue diagnosis essential. We report a 49-year-old man who presented with fever, epistaxis, and multifocal nodular and ulcerated skin lesions involving the right wrist, chest, and scalp, as well as gingival hyperplasia. Skin biopsy was reported to confirm cutaneous myeloid sarcoma, consistent with leukemia cutis in the setting of AML. Histopathology demonstrated dense dermal infiltration by atypical myeloid cells with relative epidermal sparing. The patient was diagnosed with AML with cutaneous involvement and was started on systemic chemotherapy. This case emphasizes the importance of early biopsy of suspicious skin lesions in patients with known or suspected hematologic malignancy and highlights the adverse prognostic implications of leukemia cutis.

## Introduction

Leukemia cutis (LC) refers to clinically apparent cutaneous infiltration by leukemic cells and may precede, occur concurrently with, or follow the diagnosis of systemic leukemia [1-2]. In acute myeloid leukemia (AML), cutaneous involvement is often described as cutaneous myeloid sarcoma, reflecting extramedullary accumulation of myeloid blasts within the skin [1,3].

The diagnosis is challenging because the lesions are protean and can present as papules, nodules, plaques, tumors, ulcers, or diffuse erythematous eruptions that resemble more common dermatologic conditions [1-2,4-5]. Recognition is clinically important because LC in AML has been associated with additional extramedullary disease and inferior survival outcomes [6-7]. We present a biopsy-confirmed case of cutaneous myeloid sarcoma/LC at the time of AML diagnosis and discuss its clinicopathologic significance in the context of the published literature.

## Case presentation

A 49-year-old previously healthy man presented with fever and epistaxis and was found to have newly diagnosed AML prior to the initiation of chemotherapy. His medical history was otherwise unremarkable, with no comorbidities, no regular medication use, and no family history of cancer. He worked as a teacher.

**Figure 1:**
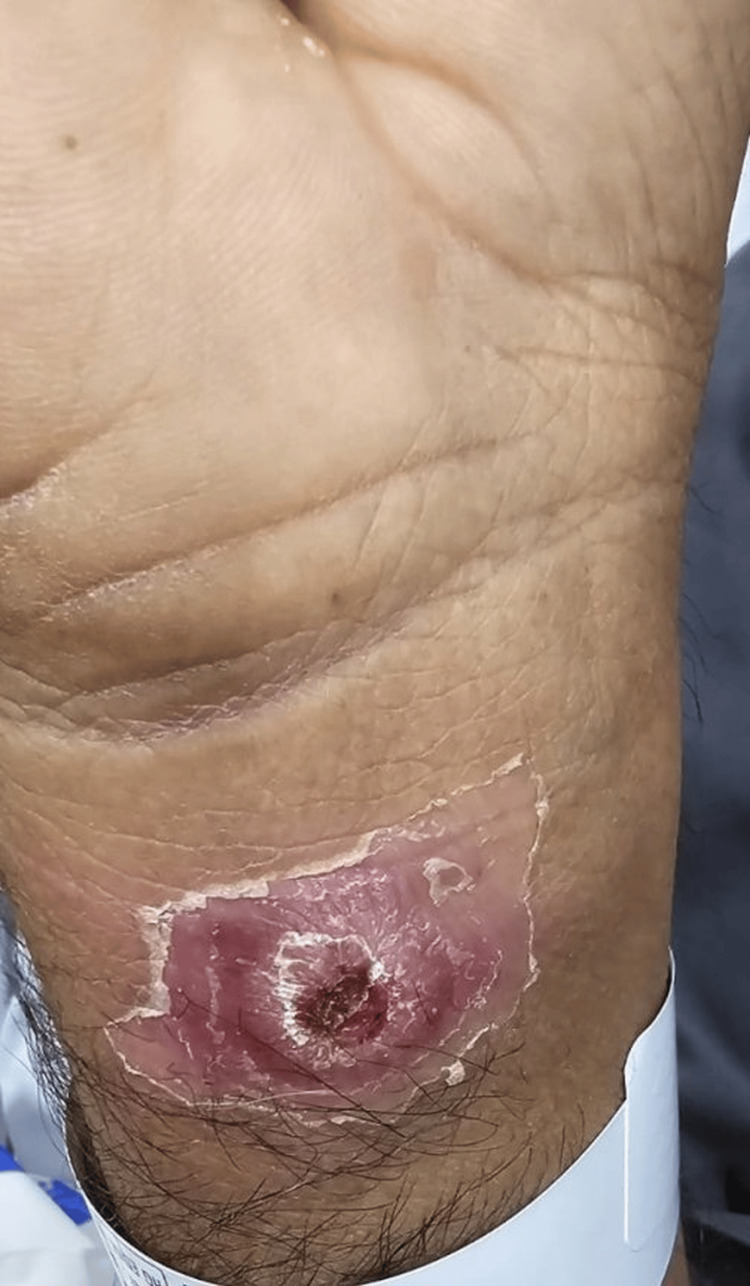
Well-defined ulcerated lesion over the right wrist with scaly margins, peeling, and a surrounding erythematous border.

**Figure 2:**
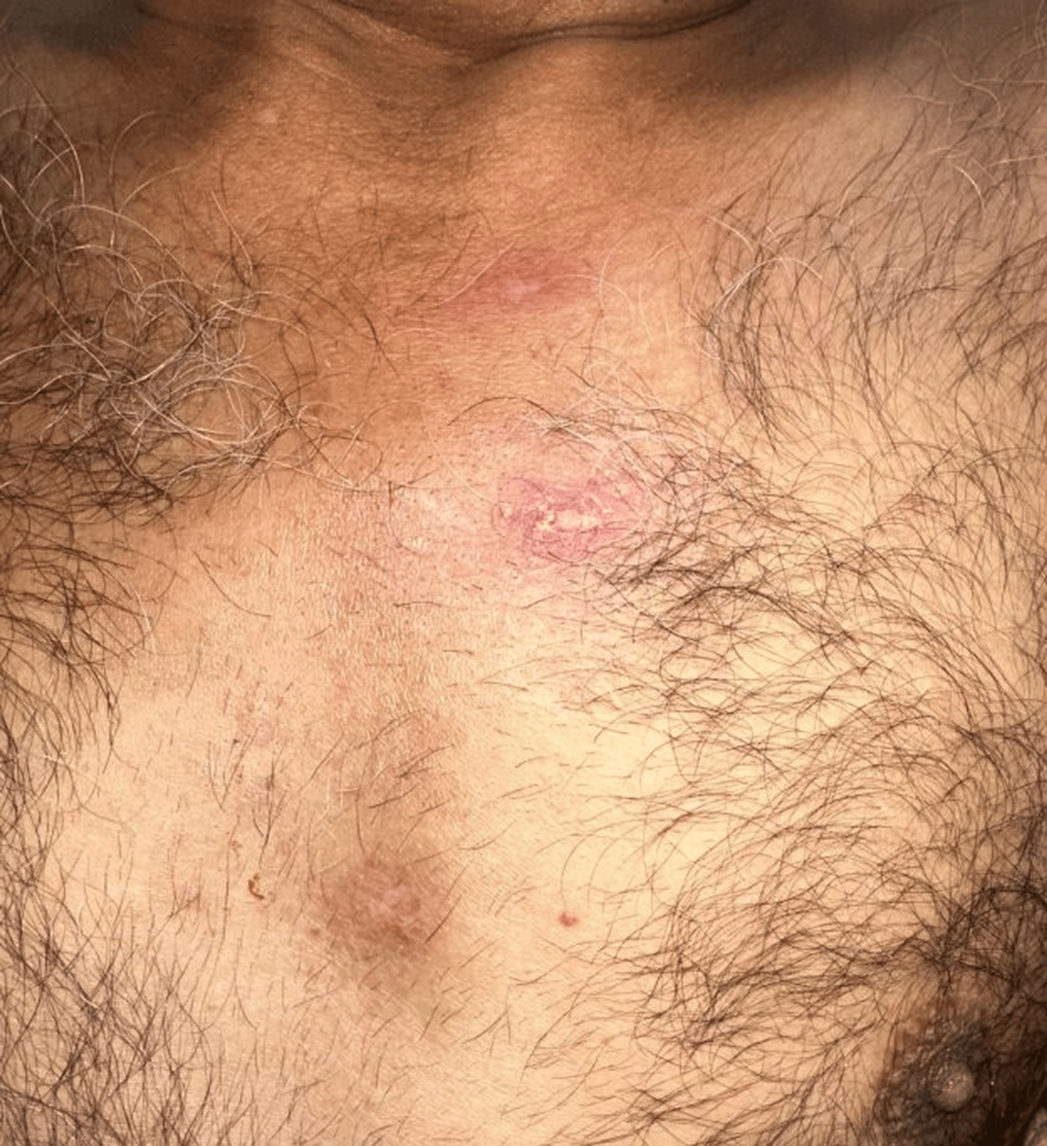
Slightly raised erythematous chest lesion with a smooth surface and mild scaling.

**Figure 3:**
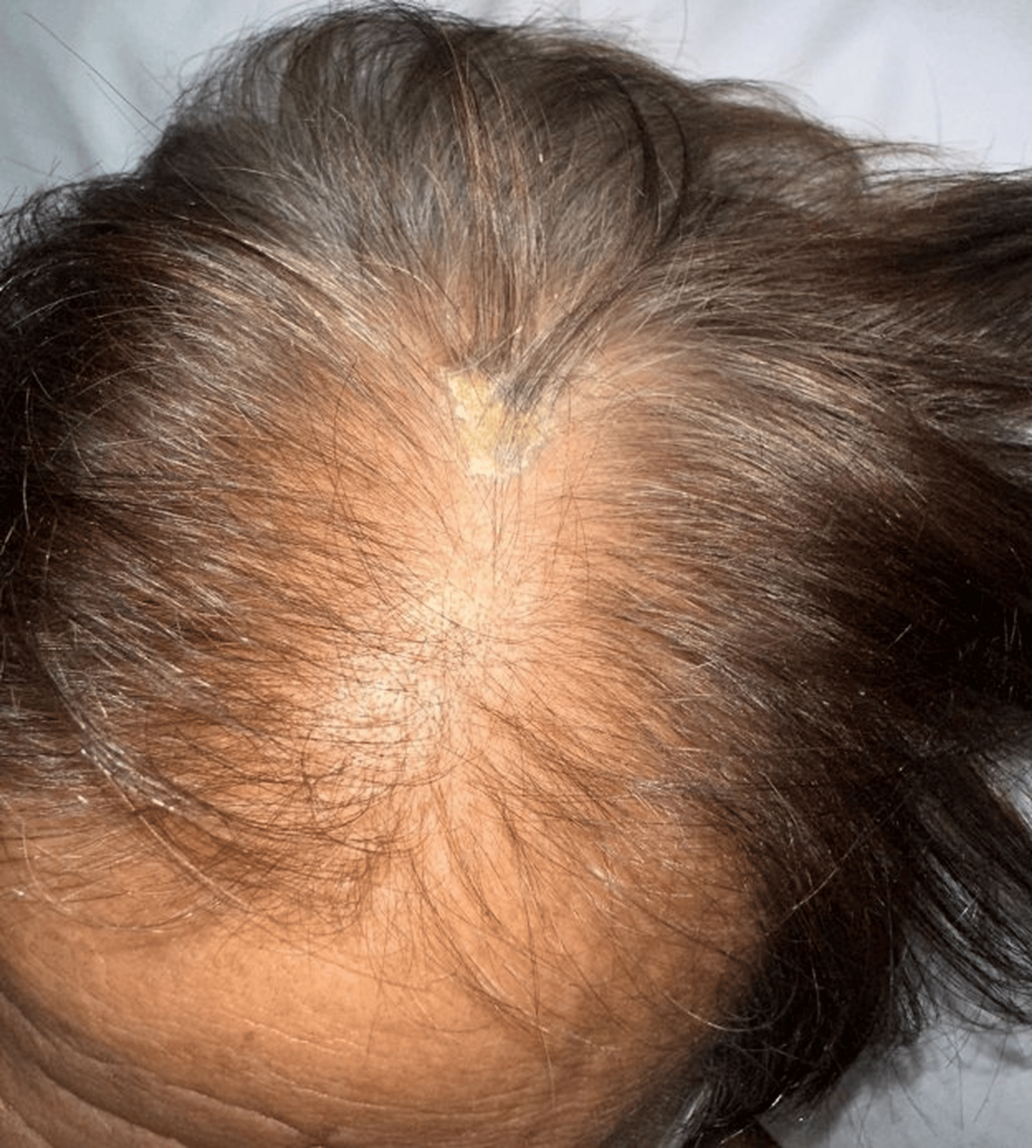
Localized scalp lesion with yellowish crusting and surrounding erythema.

**Figure 4:**
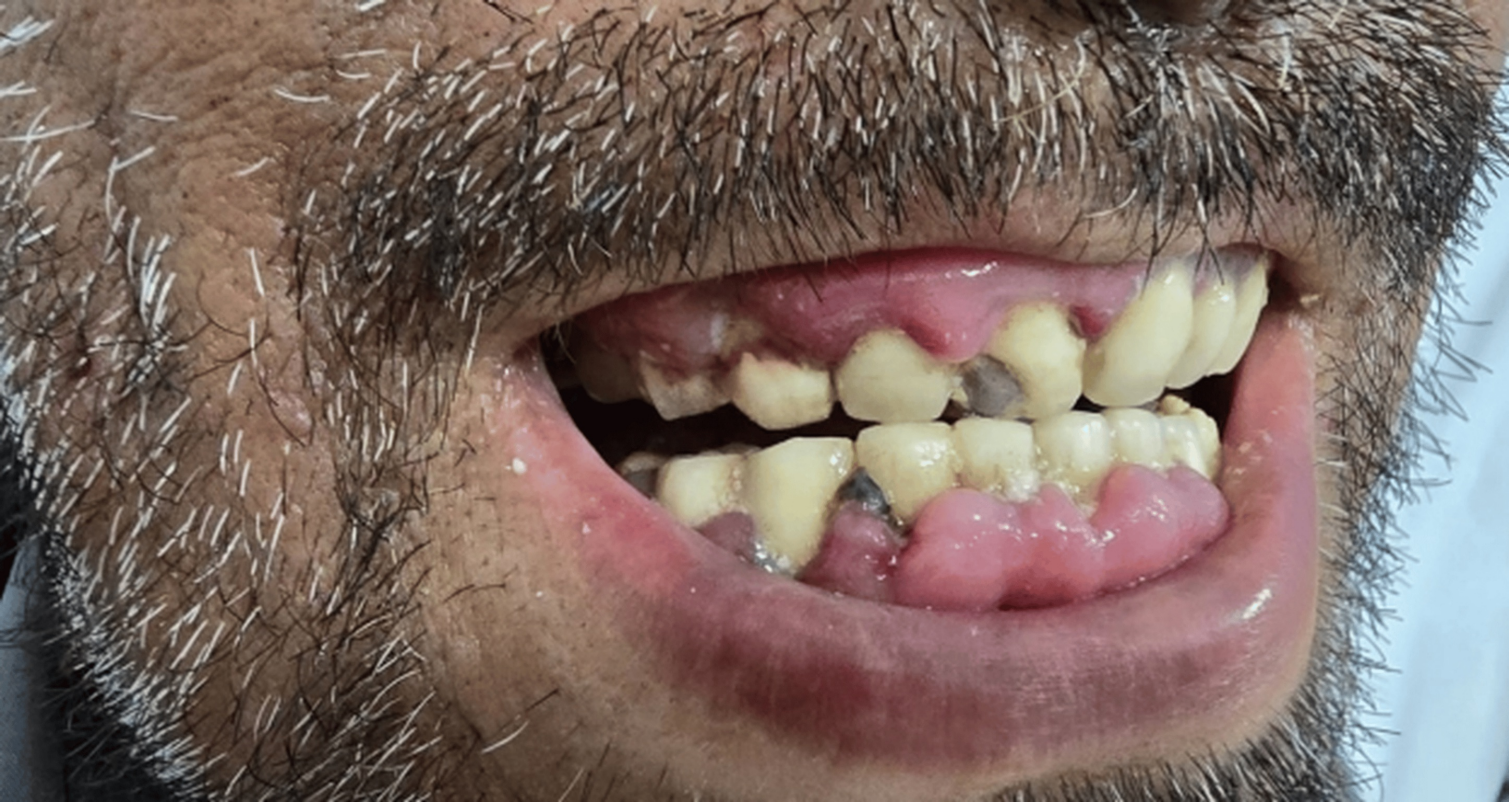
Gingival swelling and erythema.

**Figure 5:**
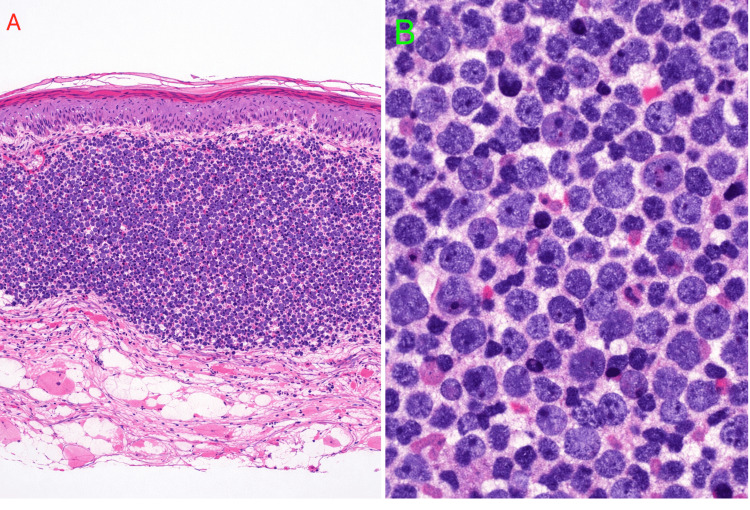
Skin biopsy demonstrating cutaneous myeloid sarcoma/leukemia cutis in acute myeloid leukemia. A: Low-power view showing dense diffuse dermal infiltration by leukemic cells with relative sparing of the epidermis. B: High-power view showing atypical myeloid cells with a high nuclear-to-cytoplasmic ratio, irregular nuclei, fine chromatin, and conspicuous nucleoli.

On physical examination, he had multiple cutaneous lesions involving the right wrist, chest, and scalp (Figures 1-3), in addition to gingival hyperplasia (Figure 4). The lesions were nodular, ulcerated, punctate, and scaly in appearance. Given the setting of untreated newly diagnosed AML, these findings were concerning for LC. Histopathologic examination of a skin biopsy confirmed cutaneous myeloid sarcoma, consistent with leukemic cutaneous involvement (Figure 5). He was subsequently treated with induction chemotherapy using 7+3 (cytarabine and daunorubicin), with quizartinib added because of FLT3-ITD-mutated AML.

## Discussion

This case illustrates several features that make LC diagnostically important and clinically consequential in AML. First, the lesions were morphologically heterogeneous, with ulceration on the wrist, erythematous papules or plaques on the chest, crusted scalp involvement, and concurrent gingival hyperplasia. This broad clinicomorphologic spectrum is well described in the literature and explains why LC may initially be mistaken for infection, vasculitis, drug eruption, inflammatory dermatoses, or secondary skin malignancy [1-2,4-5].

Second, this case underscores the central role of skin biopsy. In patients with known or suspected leukemia, biopsy should be pursued promptly when new or unexplained cutaneous lesions appear, especially when they are multifocal or progressive [2,4,8]. Typical histopathologic findings include a diffuse dermal and/or subcutaneous infiltrate of atypical hematopoietic cells, often with relative epidermal sparing, as seen in our patient [4,8-9]. In our patient, the biopsy diagnosis established the presence of cutaneous myeloid sarcoma and directly linked the skin findings to the underlying AML.

Third, this case is clinically relevant because LC generally reflects aggressive disease biology and has historically been associated with inferior survival in AML. More recent literature, however, suggests that prognosis should now be interpreted in the context of contemporary AML therapy. In particular, outcomes in FLT3-mutated AML have improved with the incorporation of tyrosine kinase inhibitors; in the phase 3 QuANTUM-First trial, quizartinib added to standard chemotherapy improved overall survival in newly diagnosed FLT3-ITD AML. Nevertheless, data specifically showing that these advances offset the adverse prognostic impact of LC remain limited, so LC should still be regarded as a high-risk extramedullary manifestation that warrants prompt AML-directed systemic therapy [1,6-7].

Management of cutaneous myeloid sarcoma is primarily systemic rather than skin-directed. Current literature supports treating LC in the context of AML-directed therapy, because local control alone does not address the underlying hematologic disease [3,10-11]. Radiotherapy may be considered for isolated, symptomatic, bulky, or refractory lesions, but it is generally an adjunct rather than a substitute for systemic treatment [1,3,10]. In our patient, recognition of the skin lesions as LC prompted appropriate chemotherapy initiation rather than treatment as a benign or purely dermatologic process. In addition, our patient harbored an FLT3-ITD mutation, which may be relevant to the development of extramedullary disease. Emerging evidence suggests that FLT3-ITD may influence leukemic blast trafficking through interaction with the CXCR4/CXCL12 axis, thereby potentially facilitating migration to extramedullary sites. However, the relationship is not fully established, and recent pooled data in myeloid sarcoma did not show a statistically significant enrichment of FLT3-ITD compared with AML without myeloid sarcoma. Accordingly, in this case, FLT3-ITD may have contributed biologically to the extramedullary presentation, but the association should be interpreted cautiously [12-13].

In the context of published literature, this case highlights three clinically important points. First, cutaneous myeloid sarcoma may be present at the initial diagnosis of AML and may involve multiple anatomic sites simultaneously. Second, histopathologic confirmation is essential because gross clinical morphology alone is insufficient for definitive diagnosis. Third, LC carries adverse prognostic implications and supports prompt AML-directed therapy [1-3,6-8,10-11].

## Conclusions

LC should be considered in any patient with known or suspected AML who develops new, unexplained, or polymorphic skin lesions. This case demonstrates that biopsy-confirmed cutaneous myeloid sarcoma can be the key diagnostic finding linking skin lesions to systemic leukemia. Early tissue diagnosis, correlation with the hematologic picture, and prompt initiation of AML-directed therapy are essential, as cutaneous involvement is frequently associated with aggressive disease and poor outcomes.
